# The impact of sterility-mortality tolerance and recovery-transmission trade-offs on host–parasite coevolution

**DOI:** 10.1098/rspb.2023.2610

**Published:** 2024-02-21

**Authors:** Prerna Singh, Alex Best

**Affiliations:** ^1^ Department of Ecology and Evolutionary Biology, Princeton University, Princeton, NJ 08648, USA; ^2^ School of Mathematics and Statistics, University of Sheffield, Sheffield S3 7RH, UK

**Keywords:** coevolution, trade-off, recovery, adaptive dynamics, branching

## Abstract

Understanding the coevolutionary dynamics of hosts and their parasites remains a major focus of much theoretical literature. Despite empirical evidence supporting the presence of sterility-mortality tolerance trade-offs in hosts and recovery-transmission trade-offs in parasites, none of the current models have explored the potential outcomes when both trade-offs are considered within a coevolutionary framework. In this study, we consider a model where the host evolves sterility tolerance at the cost of increased mortality and the parasite evolves higher transmission rate at the cost of increased recovery rate (reduced infection duration), and use adaptive dynamics to predict the coevolutionary outcomes under such trade-off assumptions. We particularly aim to understand how our coevolutionary dynamics compare with single species evolutionary models. We find that evolutionary branching in the host can drive the parasite population to branch, but that cycles in the population dynamics can prevent the coexisting strains from reaching their extremes. We also find that varying crowding does not impact the recovery rate when only the parasite evolves, yet coevolution reduces recovery as crowding intensifies. We conclude by discussing how different host and parasite trade-offs shape coevolutionary outcomes, underscoring the pivotal role of trade-offs in coevolution.

## 1. Introduction

Coevolution is a key driver in shaping the structure of host–parasite interactions. Much of the theoretical literature assumes that the hosts and parasites evolve in isolation. However, in natural systems, the long-term behaviour of disease interactions is linked to the interplay between host and parasite evolutionary characteristics, i.e. the coevolutionary dynamics [1,2]. Existing coevolutionary models have primarily focused on the coevolution of host resistance and parasite infectivity, either in an evolutionary invasion framework [3–10] or in a gene-for-gene/matching-allele framework [11–17]. Despite an increasing number of experimental and theoretical studies exploring host–parasite coevolution [18–21], further research is required to comprehend how various attributes of host–parasite interactions may impact coevolutionary dynamics. In particular, since most evolutionary theory examining tolerance is host-centric, there is a definite need to acknowledge tolerance in a coevolutionary framework [22,23].

Resistance in evolutionary models is usually defined as the host’s ability to act against the pathogens and reduce their fitness [24–26], while tolerance is defined as the host’s ability to limit the damage caused by parasitic infection without negatively affecting their fitness [27–29]. The number of coevolutionary invasion models which investigate host tolerance evolution [6,10,22,30], is lower than those considering host resistance [3–5,8,22,31]. Furthermore, these studies have examined the evolution of one type of tolerance, either sterility or mortality, but not both. A recent evolutionary model, based on a trade-off between the host’s sterility and mortality tolerance, investigated stable investments in both strategies and the potential for evolutionary branching under diverse ecological conditions [32]. Theoretical studies suggest hosts adjust resource allocation between reproduction and survival post-infection, implying a negative correlation between host responses to parasitic implications on fecundity and mortality [33,34]. A few empirical studies also support the existence of such a trade-off [35–37]. For instance, Pike *et al.* [37] revealed a negative correlation between population-level mortality and fecundity investment in wild-type *N*2 strains of the nematode host *Caenorhabditis elegans* exposed to the parasite *Staphylococcus aureus*, indicating a fecundity-mortality trade-off.

Meanwhile, parasite evolution within a coevolutionary framework is predominantly looked at under the assumption of a transmission-virulence trade-off [3–6,9,22]. Alizon [38] claimed that such a parasite trade-off fails to recognize the evolution of sublethal parasite effects, and taking recovery as the primary selection pressure for the parasite (instead of virulence) can address this problem. They showed how a trade-off between parasite transmission and recovery can emerge from within-host dynamics if immune activation is allowed to depend on the parasite’s growth rate. Both transmission-virulence and transmission-recovery trade-offs follow from an underlying idea that by increasing its host exploitation strategy, the parasite evolves a higher transmission rate but also reduces the infection duration [38,39]. Following this, Greischar *et al.* [40] developed a data-driven model focusing on malaria parasites to study the evolutionary impact of ecology on transmission investment, considering host recovery as a main driver of parasite evolution. They found that a positive correlation between transmission and recovery creates a strong selection pressure for parasite proliferation (trait influencing disease severity and spread) at the expense of transmission. A few more studies acknowledged the emergence of trade-offs between transmission and recovery in their models but did not analyse the trade-off itself [41–43]. Empirical evidence supports this trade-off, seen in the context of dengue virus and host immune response [44], and in a study on Zika virus transmission via mosquito bites to mice, where more infectious bites resulted in a shorter infection duration [45]. While empirical evidence supports trade-offs between sterility-mortality tolerance in hosts and transmission-recovery in parasites, no current models explore both trade-offs within a coevolutionary framework. Furthermore, we found no empirical study investigating both trade-offs in a host–parasite system. As many real-life systems exhibit coevolutionary dynamics, it is crucial to assess the applicability of results from purely evolutionary models. Our model, based on a sterility-mortality tolerance trade-off in the host and a transmission-recovery trade-off in the parasite, examines the interplay between both species in coevolution. Our work addresses this gap and highlights the need for empirical research to validate and refine our coevolutionary model, enhancing our understanding of complex ecological relationships.

Furthermore, existing coevolutionary models are based on one of two key assumptions: (i) each evolving trait is controlled solely by either the host or the parasite, with no interaction between the two [3,46], or (ii) both host and the parasite share control over an evolving epidemiological trait [4–6,8,10,14,22,30]. In the latter case, the evolving trait therefore depends upon the combined investment levels of both host and the parasite rather than their specific strategies. Most of these models considered transmission and virulence to be the traits controlled by both host and parasite [5,6,8,10,14,22,30]. In this study, we assume both the host and parasite evolve their traits without sharing control over a common epidemiological trait.

Our analysis starts by examining the stable investment levels of the host and the parasite to explore how ecological factors affect coevolution. We then investigate the potential for diversity through the coevolutionary process, either due to cycles or through the coexistence of multiple host and parasite strains. Additionally, we examine how various host and parasite trade-off shapes influence coevolutionary outcomes, aiming to identify differences between coevolution and independent host–parasite evolution.

## 2. Model and methods

We investigate the coevolutionary dynamics of a host–parasite system using a generic susceptible-infected-susceptible (SIS) model framework [47]. Extending the host evolutionary model studied by Singh & Best [32], we assume that the host evolves both sterility and mortality tolerance and the two tolerance components are related by a trade-off function. Additionally, the parasite coevolves with the host and follows a transmission-recovery trade-off. As such, the parasite can increase its transmissibility but at the cost of a reduced infectious period due to increased recovery. Our model is particularly suited for scenarios involving asexual reproduction and is a good fit for systems characterized by horizontal parasite transmission, such as the plant *Arabidopsis thaliana* infected by *Cucumber mosaic virus* (CMV) [35]. We further consider a density-dependent birth rate and a homogeneous, well-mixed host population. The following equations describe the population dynamics of the ceoevolutionary model where *X* and *Y* denote the densities of susceptible and infected hosts, respectively:

2.1 $$\begin{array}{rl} \frac{dX}{dt} & =(a-qN)(X+fY)-\beta XY-bX+\gamma Y\\ \mathrm{and}\;\frac{dY}{dt} & =\beta XY-((\alpha -\tau )+b+\gamma )Y. \end{array}\}$$

Here *N* = *S* + *I*, and the parameters are detailed in table 1. The birth rate of all hosts is *a*, but infection diminishes the reproduction of infected hosts by a factor of *f*, where the value of *f* indicates the relative birth rate of infected hosts (0 < *f* < 1). The natural death rate of all hosts is *b*, and *q* represents the impact of crowding on overall host birth rate. Susceptible hosts can get infected by a mass-action transmission process with coefficient *β*. *α* represents the additional death rate resulting from parasitic infection, while *τ* reflects the host’s tolerance to parasite-induced mortality, leading to a reduction in *α* (i.e. *τ* < *α*). Finally, *γ* is the rate at which infected hosts recover from the infection and become susceptible to infection again.

**Table 1.** Description of parameters.

parameters	definition	default value
*a*	host birth rate	2.5
*b*	host natural death rate	0.05
*q*	crowding effect	0.5
*α*	disease-induced mortality rate (virulence)	2
*β*	infection transmission coefficient	varies
*γ*	recovery rate of infected hosts	varies
*f*	sterility tolerance	varies
*τ*	mortality tolerance	varies
*τ*′(*f* *)	host trade-off gradient	–1.3996
*β*′(*γ* *)	parasite trade-off gradient	0.97561

Additionally, we assume that the traits of sterility and mortality tolerance (*f* and *τ*) are under selection in the host, while the transmission and recovery rates (*β* and *γ*) are parasite-driven traits. High/low values of *f* and *τ* indicate higher/lower investment in respective tolerance traits. Similarly, high/low values of *β* and *γ* indicate higher/lower values of parasite transmission and recovery rate, respectively. We chose our parameters in a way that the parasite persists in the system at an endemic equilibrium.

### (a) Modelling within the adaptive dynamics framework

We model the coevolution of the host and parasite using the classic adaptive dynamics framework [48–51]. We assume that the resident host strain with strategy (*f*, *τ*) and the resident parasite strain with strategy (*γ*, *β*) have reached a stable, positive equilibrium. We then determine the invasion fitness of a mutant host strain with strategy (*f*_*m*_, *τ*(*f*_*m*_)) or a mutant parasite strain with strategy (*γ*_*m*_, *β*(*γ*_*m*_)) that are attempting to invade the resident equilibrium. The expression for the mutant host fitness is given by:

2.2 $$s(f,f_{m},\gamma )=\frac{Tr+\sqrt{Tr^{2}-4\mathrm{Det}}}{2},$$

where Tr = (*a* − *q*(*X* * + *Y* *) − *b* − *β*(*γ*)*Y* *) − (*α* + *b* + *γ* − *τ*(*f*_*m*_)), and Det = (*a* − *q*(*X* * + *Y* *) − *b* − *β*(*γ*)*Y* *)(*τ*(*f*_*m*_) − *α* − *b* − *γ*) − *β*(*γ*) *Y* *(*a f*_*m*_ − *q f*_*m*_(*X* * + *Y* *) + *γ*) are the trace and determinant of the host mutant dynamics system (refer to the electronic supplementary material, appendix for more details, also see Best [4]). On the other hand, the parasite invasion fitness is given by:

2.3 $$r(\gamma_{m},\gamma ,f)=\beta (\gamma_{m})X^{*}-(\alpha +b+\gamma_{m}-\tau (f)).$$

Here, *X* * and *Y* * are the susceptible and infected population densities evaluated at the resident equilibrium, and *τ*(*f*) and *β*(*γ*) denote the respective host and parasite trade-offs. Assuming that mutations are small and rare, the coevolutionary dynamics of the host and parasite traits *f* and *γ* over evolutionary time *T* can be approximated by the following pair of equations:

2.4 $$\frac{df}{dT}\propto \phi_{h}X^{*}\frac{\partial s}{\partial f_{m}}{|}_{\;f_{m}=f}$$

and

2.5 $$\frac{d\gamma }{dT}\propto \phi_{\;p}Y^{*}\frac{\partial r}{\partial \gamma_{m}}{|}_{\gamma_{m}=\gamma },$$

where *ϕ*_*h*_ and *ϕ*_*p*_ are constants controlling the mutation speed of the host and parasite, respectively, incorporating variation in the occurrence of mutations and the mutant trait distributions [52]. For mathematical ease, we set *ϕ*_*h*_ = *ϕ*_*p*_ = 1 but later relax this assumption in the simulations. Then the host and parasite coevolve along their respective fitness gradients, $\partial s/\partial f_{m}{|}_{\;f_{m}=f}$ and $\partial r/\partial \gamma_{m}{|}_{\gamma_{m}=\gamma }$, until a coevolutionary singular point is attained where the two gradients become simultaneously zero [51,53]. Depending upon the signs of the second-order derivatives of the fitness expressions and speed of evolution of both species, we observe the following coevolutionary outcomes: (i) a continuously stable strategy (co-CSS) where both host and parasite invest in optimal levels of investment, (ii) evolutionary branching in either or both species, (iii) coevolutionary cycles and (iv) maximization/minimization of the evolving host and/or parasite traits to their physiological bounds. Additional details of these methods are given in the electronic supplementary material.

### (b) Host and parasite trade-offs

To conduct our coevolutionary analysis, we assume that the host evolves two tolerance strategies that are inversely linked by a trade-off function. Consequently, investing more resources in one tolerance strategy would come at the expense of reduced investment in the other. Meanwhile, the parasite can evolve increased infection transmission, but this leads to an increment in the host recovery rate, which indirectly incurs a cost in terms of a reduced infectious period for the parasite. We consider generic trade-off forms for both host and parasite as developed by Kisdi [50] and further elaborated by Hoyle *et al.* [54] as follows:

$$\begin{array}{rl} & \tau (f)=\tau (f^{*})-\frac{\tau^{\prime }{\left(f^{*}\right)}^{2}}{\tau^{\prime \prime }(f^{*})}(1-\;e^{\tau^{\prime \prime }(f^{*})(f-f^{*})/\tau^{\prime }(f^{*})})\\ \mathrm{and}\; & \beta (\gamma )=\beta (\gamma^{*})-\frac{\beta^{\prime }{\left(\gamma^{*}\right)}^{2}}{\beta^{\prime \prime }(\gamma^{*})}(1-e^{\beta^{\prime \prime }(\gamma^{*})(\gamma -\gamma^{*})/\beta^{\prime }(\gamma^{*})}). \end{array}$$

Here, primes denote the derivatives and (*f* *, *τ*(*f*)*) and (*γ* *, *β*(*γ* *)) are the singular strategies for the host and parasite, respectively. The choice of such a trade-off form allows us to fix the singular strategy at a chosen point and then find the slope and curvature of the trade-off function at that chosen strategy. Here, we fix the host singular strategy at (0.5, 1) and the parasite strategy at (1, 2). Then slopes *τ*′(*f* *) and *β*′(*γ* *) are calculated such that the singularities occur at these chosen points (i.e. host and parasite fitness gradients become zero at *f* * and *γ* *, respectively). The curvatures *τ*″(*f* *) and *β*″(*γ* *), can be chosen as per the requirement for an accelerating or decelerating cost function. The graphical representation of both trade-offs is given in the supplementary file. The concave shape implies increasing costs in strategies (*τ*″(*f* *) < 0, *β*″(*γ* *) < 0), while the convex shape indicates decelerating costs (*τ*″(*f* *) > 0, *β*″(*γ* *) > 0).

## 3. Results

Initially, we assume accelerating trade-offs for both the host and parasite, which typically results in stable investments at a coevolutionary stable strategy (co-CSS), as noted by Hoyle *et al.* [54]. This assumption suggests that investing more in tolerance to parasite-induced sterility for the host and higher transmission for the parasite will become progressively more costly. Throughout this study, we only get one singular strategy corresponding to a particular parameter set and the trade-off shape.

**Figure 1.**
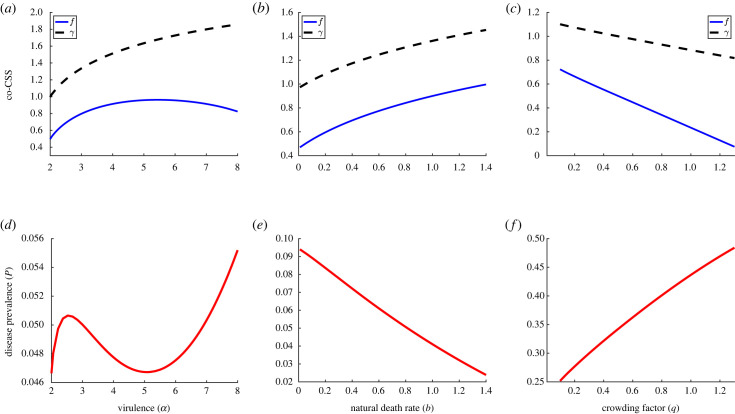
Variation in co-CSS strategies along with varying (*a*) virulence *α*, (*b*) natural death rate *b* and (*c*) crowding factor *q*. (*d*–*f*) Corresponding disease prevalence plots. Here, we use trade-offs with accelerating costs: *τ*″(*f* *) = −1.5 and *β*″(*γ* *) = −1.5, and remaining parameters are the same as in table 1.

To reveal how ecological and epidemiological factors drive coevolution, we present the variation in co-CSS points for the disease-induced mortality rate *α*, intrinsic death rate *b* and crowding factor *q* (figure 1). We found that host investment in sterility tolerance is maximized at intermediate virulence, but that the recovery rate increases monotonically as virulence increases (figure 1*a*). This means that the host can benefit from increasing infected reproduction as long as the virulence is not too high and infected hosts live long enough to reproduce. However, extreme levels of virulence means infected hosts are dying quickly and will reduce the benefit of sterility tolerance as a host strategy. The parasite, on the other hand, experiences selection for higher recovery rate, and therefore higher transmission, as virulence increases (figure 1*a*). This is similar to classic results showing increased mortality leads parasites to evolve higher transmission as it is better to infect quickly when the infectious period is small (see electronic supplementary material, appendix).

In addition, we present the corresponding disease prevalence plots since the feedback to host and parasite selection is directly linked to the prevalence of the parasite in the system (figure 1*d*–*f*). The parasite prevalence is given by the formula *P* = *Y* */(*X* * + *Y* *), where *X* * and *Y* * are the susceptible and infected hosts’ densities at the corresponding co-CSS points. The prevalence initially increases with *α*, then experiences a sharp decline for intermediate virulence, and finally increases again as virulence becomes excessively high (figure 1*d*). This is intriguing because high levels of virulence should lead to lower prevalence due to the increased mortality of infected hosts, but as the parasite coevolves, the combined impact of high infection transmission and high birth rates at that point overshadows the effect of high virulence. Our prevalence patterns closely align with the trends of mortality tolerance throughout the study, similar to what is generally predicted by purely evolutionary models [28,29].

As natural death rate *b* increases, both host fecundity tolerance *f* and recovery rate *γ* increase rapidly (figure 1*b*). A high death rate leads to reduced parasite prevalence by lowering the lifespan, as confirmed by figure 1*e*. Therefore, when the death rate is high, an investment in mortality tolerance, which acts to lengthen the infectious period, will be a less beneficial strategy. This will reduce selection for mortality tolerance as a host strategy and consequently lead to higher fecundity tolerance due to the trade-off; the host can balance out the greater number of deaths by increasing reproduction through increased selection for fecundity tolerance. On the other hand, since the parasite’s infectious period is short due to its high intrinsic death rate, it benefits more by increasing its transmission, which leads to higher recovery via trade-off (as shown in figure 1*b*). Again, these patterns match the purely evolutionary trends (refer to electronic supplementary material, appendix).

Next, we have the co-CSS pattern corresponding to the increasing crowding effect *q* (figure 1*c*). We get strictly decreasing host and parasite investments in their respective strategies, where the decline is sharper for the host strategy *f*. As crowding increases, the overall birth rate diminishes and lowers the benefit of sterility tolerance as a host fitness strategy, hence decreasing it. On the contrary, we found the disease prevalence to continuously increase as crowding increased (figure 1*f*). This is in contrast to the pattern from the host-only evolution model [32], where prevalence was a decreasing function of crowding, with or without evolution. This suggests that the coevolution of the parasite creates a reverse feedback on prevalence, thus increasing it. In particular, the increased prevalence driven by the host strategy (high mortality tolerance) results in a reduced need for investment in transmission as a parasite strategy. Instead, the parasite can benefit more from a longer infectious period through a lowered recovery rate as crowding increases, as shown in figure 1*c*. While the pattern for sterility tolerance remains the same as in the host-only evolution scenario, varying crowding creates no impact on the recovery rate when the parasite evolves in isolation (see electronic supplementary material, appendix). Thus, it is evident that coevolution can significantly alter the co-CSS trends.

### (a) Coevolutionary outcomes

To better understand the various coevolutionary outcomes, we performed numerical simulations following the algorithm outlined in the electronic supplementary material, appendix of Best *et al.* [5]. We consider 30 strains of each host and parasite species and set all densities except one of each strain (one host, one parasite) to 0. Then we numerically solve the population dynamics for a time sufficient for the populations to reach their attractor. We allow the host and parasite to mutate with equal probabilities; mutant strains are generated by small deviations nearby the current traits. The population dynamics are then solved again for a further time and strains with densities below a set threshold (0.001) are considered to be extinct and removed. We also introduce an approximate demographic stochasticity function which determines the probability of initial invasion success of the mutant. Specifically, mutant strains characterized by negative or marginal initial growth rates are considered to be extinct. This function ensures that the relative speeds of evolution of both coevolving species match the analytical framework, as it guarantees that the probability of a successful mutant invasion is directly proportional to its fitness, as outlined in previous work [52]. This process is then repeated to follow the directional evolution of both species (see Best *et al.* [6] and Boots *et al.* [8] for more details on the simulation process). The framework with *m* types of each host and parasite species is given by the following equations:

3.1 $$\begin{array}{rl} \frac{dX_{h}}{dt} & =(a-q(\sum_{h=1}^{m}X_{h}+\sum_{\;p=1}^{m}\sum_{h=1}^{m}Y_{hp}))(X_{h}+f_{h}\sum_{\;p=1}^{m}Y_{hp})\\ & \;-X_{h}\sum_{\;p=1}^{m}\beta_{\;p}\sum_{h=1}^{m}Y_{hp}-bX_{h}+\sum_{\;p=1}^{m}\gamma_{\;p}Y_{hp}\\ \mathrm{and}\;\frac{dY_{hp}}{dt} & =X_{h}\beta_{\;p}\sum_{h=1}^{m}Y_{hp}-(\alpha -\tau_{h}+b+\gamma_{\;p})Y_{hp}, \end{array}\}$$

where *X*_*h*_ represents the density of susceptible hosts of type *h* and *Y*_*hp*_ represents the infected hosts of type *h* infected by parasite type *p*. Here, *f*_*h*_, *τ*_*h*_, *γ*_*p*_ and *β*_*p*_ are the respective host and parasite traits, obeying their respective trade-offs. The parameters and evolving traits are the same as for the one host–one parasite system (2.1). We further discuss the range of qualitative outcomes that can occur depending upon the trade-off shapes and speed of mutation in both species.

#### (i) Evolutionary branching

**Figure 2.**
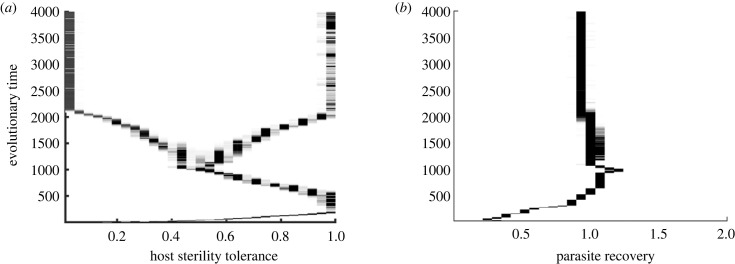
Simulations showing branching in the (*a*) host strategy *f*, while (*b*) parasite strategy *γ* evolves to its CSS over evolutionary time, when both species have equal mutation speeds, i.e. *ϕ*_*h*_ = *ϕ*_*p*_. Here, we consider a weakly decelerating host trade-off with *τ*″(*f* *) = 0.05 and weakly accelerating parasite trade-off with *β*″(*γ* *) = −0.15.

Similar to the findings of Singh & Best [32], we found that the host population can branch in our coevolutionary set up for a limited selection of weakly decelerating host trade-off curves while the parasite (initially) remains at its CSS (figure 2). It is well understood that for the occurrence of a branching point, the singular strategy must be CS (convergent stable strategy which attracts nearby strategies), but not ES (evolutionary stable strategy which cannot be invaded by nearby mutants). In addition, the concerned species must satisfy the condition of mutual invadibility (i.e. *M*_*h*_ = ∂^2^ *s*/∂*f*∂*f*_*m*_ < 0 or *M*_*p*_ = ∂^2^ *r*/∂*γ*∂*γ*_*m*_ < 0), where *M*_*h*_ and *M*_*p*_ denote the expressions for mutual invadibility of the host and parasite, respectively [48,50]. Based on our analytical calculations, we observed that the host population meets the criteria for branching across a broad range of parameters, whereas the parasite population does not (see electronic supplementary material, appendix, figure S8). Consequently, under suitable trade-off choices, both host and parasite population will evolve towards the cosingular point but when close to the singularity, the host will branch to become dimorphic (figure 2*a*), whereas the parasite will remain monomorphic (figure 2*b*).

After the host undergoes branching, the resident population now consists of two host-one parasite strains at equilibrium. So there are two susceptible classes *X*_1_ and *X*_2_, with strategies (*f*_1_, *τ*(*f*_1_)) and (*f*_2_, *τ*(*f*_2_)) and one infected class, *Y* = *Y*_1_ + *Y*_2_, with parasite strategy (*γ*, *β*(*γ*)). The population dynamics of such a system is given by the following equations:

3.2 $$\begin{array}{rl} \frac{dX_{1}}{dt} & =(a-qN)(X_{1}+f_{1}Y_{1})-\beta X_{1}(Y_{1}+Y_{2})-bX_{1}+\gamma Y_{1},\\ \frac{dX_{2}}{dt} & =(a-qN)(X_{2}+f_{2}Y_{2})-\beta X_{2}(Y_{1}+Y_{2})-bX_{2}+\gamma Y_{2},\\ \frac{dY_{1}}{dt} & =\beta X_{1}(Y_{1}+Y_{2})-(\alpha -\tau_{1}+b+\gamma )Y_{1}\\ \mathrm{and}\;\frac{dY_{2}}{dt} & =\beta X_{2}(Y_{1}+Y_{2})-(\alpha -\tau_{2}+b+\gamma )Y_{2}. \end{array}\}$$

Please note that even though we introduce two infected types *Y*_1_ and *Y*_2_ here, there remains just one parasite with strategy (*γ*, *β*(*γ*)). Here, *τ*_1_ = *τ*(*f*_1_), *τ*_2_ = *τ*(*f*_2_) and *β* = *β*(*γ*). The expression for the mutant parasite’s growth rate (i.e. fitness) in an environment with two host strains is given by

**Figure 3.**
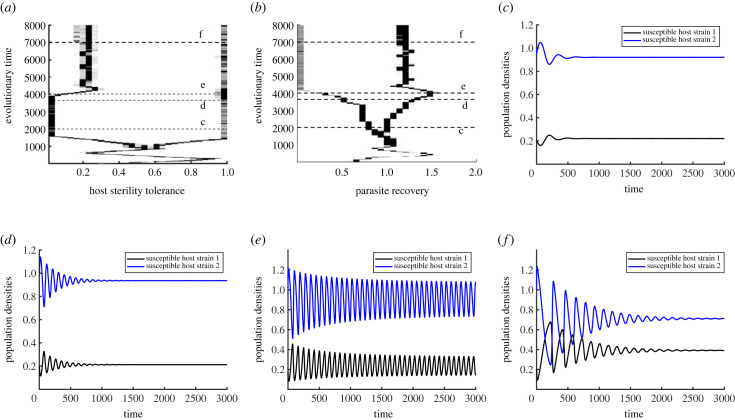
(*a*,*b*) Simulations showing branching in the parasite occurs after the host branches when *ϕ*_*h*_ = *ϕ*_*p*_. Horizontal dashed lines indicate strategies chosen at that time in the corresponding plots. (*c*–*f*) Limit cycles in the population dynamics of the two host–two parasite strain model emerge after the host branching. In (*c*), *f*_1_ = 0.01, *f*_2_ = 1, *γ*_1_ = 0.76 and *γ*_2_ = 1.05; (*d*) *f*_1_ = 0.01, *f*_2_ = 1, *γ*_1_ = 0.5 and *γ*_2_ = 1.382. (*e*) Non-damped oscillations emerge when *f*_1_ = 0.01, *f*_2_ = 1, *γ*_1_ = 0.2 and *γ*_2_ = 1.45. (*d*) Population dynamics again tends to stabilize when *f*_1_ = 0.2, *f*_2_ = 1, *γ*_1_ = 0.01 and *γ*_2_ = 1.17. The chosen trade-off curvatures are *τ*″(*f* *) = 0.1 and *β*″(*γ* *) = −0.1.

3.3 $$\begin{array}{rl} r(\gamma_{m},\gamma ,f_{1},f_{2}) & =\beta (\gamma_{m}){X_{1}}^{*}(\alpha -\tau_{2}+b+\gamma_{m})\\ & \;+\beta (\gamma_{m}){X_{2}}^{*}(\alpha -\tau_{1}+b+\gamma_{m})\\ & \;-(\alpha -\tau_{1}+b+\gamma_{m})(\alpha -\tau_{2}+b+\gamma_{m}), \end{array}$$

where $X_{1}^{*}$ and $X_{2}^{*}$ are the equilibrium densities of two host strains and are functions of (*f*_1_, *γ*) and (*f*_2_, *γ*), respectively (see electronic supplementary material, appendix for the derivation of fitness expression). We further assume that for a chosen pair of host strategies (*f*_1_, *f*_2_), the parasite will be at singular strategy (*γ* *, *β*(*γ* *)). To demonstrate the possibility of branching in the parasite within system 3.2, we numerically evaluate the values of the mutual invadibility expression *M*_*p*_ = ∂^2^ *r*/∂*γ*∂*γ*_*m*_ (and similarly for ES and CS), at its singular point. From numerical calculations, we find that the parasite fulfils the required conditions for branching under appropriate choices of trade-offs and host strategies (see electronic supplementary material, appendix, figure S8). In particular, for host strategies chosen as *f*_1_ = 0.01, *f*_2_ = 1, weakly decelerating host trade-off with *τ*″(*f* *) = 0.1 and an accelerating parasite trade-off with *β*″(*γ* *) = −0.1, the parasite strategy (*γ* *, *β*(*γ* *)) = (1, 2) is CS but not ES. Moreover, the condition of mutual invadibility holds i.e. *M*_*p*_ < 0 at the strategy. Our simulations confirm this finding, as we find examples where branching occurs in the strict order of first host, then parasite (figure 3*a*,*b*). So for a dimorphic resident host, the parasite can branch into two coexisting strains. In one of these strains, infected hosts can rapidly recover from the infection, while in the other they will persist for a longer duration. However, an unconventional pattern that we noticed for a variety of trade-off shapes is that the resulting dimorphic strains are not all extreme. This means that one of the coexisting strains in both host and the parasite settle at values different from the assumed bounds of evolution, i.e. *f* ∈ (0, 1), *γ* ∈ (0.01, 2). This result is distinctive compared with the findings of the majority of the theory which predicts that the dimorphic strains evolve to their extremes [6,22,24,25,32,55]. A possible explanation could be due to the impact of fluctuating ecological dynamics over coevolution. It is understood that population dynamics play a considerable role in the host–parasite evolution and feedbacks caused by the ecological and evolutionary interactions can lead to qualitative shifts in the evolutionary outcomes [5,56–58]. By examining our model system consisting of two hosts and two parasites (system 3.1 for *m* = 2), we were able to identify limit cycles in the population dynamics (as shown in figure 3*e*). This fluctuating behaviour in our ecological model can cause shifts in the evolutionary outcomes, affecting the coexistence of such dimorphic strains (see Best & Ashby [57] for more details).

In figure 3*c*–*f*, we demonstrate how limit cycles emerge in our two host–two parasite population dynamics system as four different sets of host and parasite strategies are chosen from simulations after host branching. As such, when the sterility tolerance of the two host strains and the parasite’s current strategy are taken at time *T* = 2000, population densities converge to the equilibrium (figure 3*c*). For strategies chosen after further evolutionary time with greater differences between the host strains’ values, we detect increasing fluctuations (figure 3*d*). At *T* = 4000 when parasite strains are even further apart, stable limit cycles occur (figure 3*e*). However, for strategies chosen after this stage, we find that the population dynamics again start to converge to the equilibrium (figure 3*f*). These plots indicate that the coevolutionary dynamics seem to settle close to the boundary of equilibria and cycles. In general, cycles in our ecological system depend predominantly upon the initial chosen values of *f* and *γ* and are more likely when the difference between strategies is higher. These trends in the population dynamics may stem from the interplay between multiple host and parasite strains, where the growth of one impacts the other, cyclically leading to phases of increase and decrease in their populations. Please note that we assumed equal mutation speeds to construct figures 2*a*,*b* and 3*a*,*b*. In reality, parasites often evolve at a significantly faster rate than their host organisms [59,60]. To explore this scenario, we conducted additional simulations assuming the parasite’s mutation speed to be 10 times that of the host, i.e. *ϕ*_*p*_ = 10*ϕ*_*h*_ (see electronic supplementary material, appendix, figure S6). We observed that when the parasite’s mutation rate is higher, populations take longer to branch and reach stable boundaries, while the key evolutionary patterns remain consistent.

#### (ii) Ecological and coevolutionary cycles/fluctuating selection dynamics

**Figure 4.**
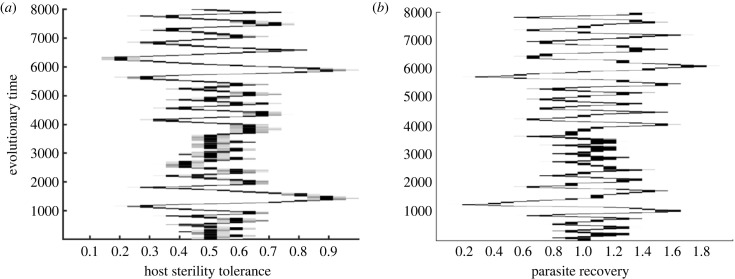
Simulation output showing the coevolutionary cycles in both host and the parasite when *ϕ*_*p*_ = 10*ϕ*_*h*_. Here, we have *τ*″(*f* *) = −0.05, *β*″(*γ* *) = 0.1 and remaining parameters are the same as in table 1.

Besides co-CSS and branching in the host and parasite populations, we discovered fluctuating selection dynamics (FSD) in the form of coevolutionary cycles (figure 4*a*,*b*). To confirm the presence of coevolutionary cycles, we performed theoretical analysis as outlined by Lehtinen & Geritz [61] (also see Best *et al.* [5] and Kisdi *et al.* [62]). For this, it is sufficient to show that parameters and trade-offs exist which produce a Hopf bifurcation (a critical point where a system’s stability switches from an equilibrium to a limit cycle). It has been proven that a sufficient condition to confirm the Hopf bifurcation is that the Jacobian matrix *J* formed of the fitness gradient equations has purely imaginary eigenvalues [61]. This occurs when systems cross the path where trace *T*(*J*) = 0 and determinant, *D*(*J*) > 0. The explicit form of the Jacobian matrix is given below:

$$J=\left(\begin{array}{cc} \phi_{h}X^{*}(\frac{\partial^{2}s}{\partial f_{m}^{2}}+\frac{\partial^{2}s}{\partial f\partial f_{m}}) & \phi_{h}X^{*}(\frac{\partial^{2}s}{\partial f_{m}\partial \gamma })\\ \phi_{\;p}Y^{*}(\frac{\partial^{2}r}{\partial \gamma_{m}\partial f}) & \phi_{\;p}Y^{*}(\frac{\partial^{2}r}{\partial \gamma_{m}^{2}}+\frac{\partial^{2}r}{\partial \gamma_{m}\partial \gamma }) \end{array}\right).$$

**Figure 5.**
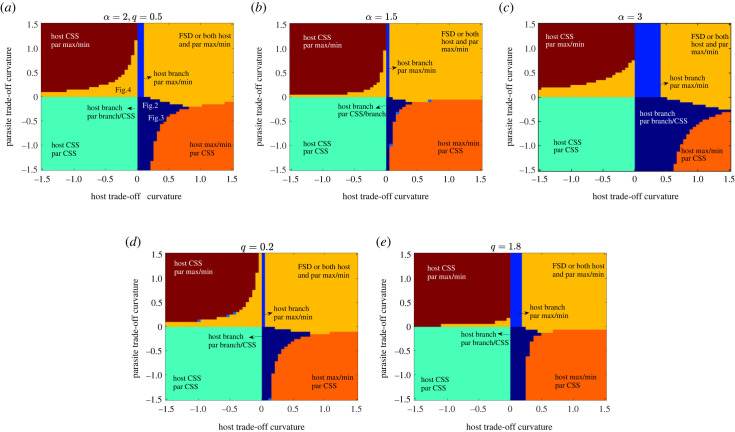
Classification of coevolutionary outcomes based on the analytical conditions as trade-off shapes vary. For each combination of host and parasite trade-off curvature, the system consisting of two fitness gradients is solved for a co-singular point. The behaviour at each co-singularity is then classified according to the theoretical conditions it satisfies. CSS, convergent singular strategy; max/min, maximization or minimization of the evolving trait; FSD, fluctuating selection dynamics or coevolutionary cycles.

Besides trade-off curvatures, the mutation speed parameters *ϕ*_*h*_, *ϕ*_*p*_ also play a significant role in determining when cycles generate/vanish and the amplitudes of fluctuations. For instance, we noted indefinite cycles when the parasite’s mutation speed was 10 times that of the host (figure 4). By contrast, equal mutation speeds could also generate cycles, but they rapidly decay, leading to branching patterns (see electronic supplementary material, appendix, figure S7). Although cycles are expected to occur for a range of trade-off shapes which satisfy *T*(*J*) > 0 and *D*(*J*) > 0, they are more likely when the host trade-off is weakly accelerating and the parasite’s trade-off is weakly decelerating. Finally, we noted from our simulations that the cycles can either occur indefinitely or be lost, resulting in different outcomes. These outcomes include stable polymorphism, or branching following the cyclic pattern but one of the two coexisting strains dies out, or both the host and the parasite evolve towards minimization or maximization (see electronic supplementary material, appendix, figure 5*a*). These irregular transitions are caused by stochastic variations between simulations, such as when cycles with small amplitude are close enough to a branching point or a repeller, or when low population densities are approximated to zero.

### (b) Impact of trade-off shapes on coevolutionary behaviour

To finally summarize how different combinations of host and parasite trade-off shapes influence the coevolutionary outcomes in this model, we categorize our simulation output for the default parameter set (figure 5*a*). We also investigate what levels of virulence *α* and crowding *q* promote the probabilities of diversification through branching and coevolutionary cycles (figure 5*b*–*e*). Here, we express the fitness equations and stability conditions as functions of the trade-off shapes at the singular strategies and classify the coevolutionary behaviour in terms of these shapes. In particular, we display the potential coevolutionary outcomes at the cosingularity (*f* * = 0.5, *γ* * = 1) for different host and parasite trade-off curvatures, *τ*″(*f* *) and *β*″(*γ*), respectively. It should be noted that these simulations do not take into account either stochasticity or mutation speeds and simply rely on the theoretical stability conditions. Therefore, the coevolutionary outcomes may differ when these factors are included. We recall that the trade-offs are accelerating when the curvatures are negative and decelerating when they are positive. In the context of weakly decelerating trade-offs, we are referring to a small positive value of the respective curvature. In all the cases, when trade-offs of both host and parasite populations are accelerating, we get a co-CSS or stable investment levels. Furthermore, a combination of weakly decelerating host trade-off and weakly accelerating parasite trade-off leads to branching in the host while the parasite evolves either to its CSS or maximizing/minimizing investment (figure 5*a*–*e*). For a similar set of trade-offs, we find that the branching in the host can lead to branching in the parasite population. In figure 5, we have classified the output in terms of the behaviour at the first singular strategy that the system attains. As branching in the parasite occurs after the host branches, we get the same signs of stability conditions for when the host branches or remains at its CSS, and thus both behaviours are represented by the same colour shades (dark blue). If either the host or parasite trade-off decelerates, we observe the respective species evolving towards maximum or minimum levels of investment, while the other species approaches its CSS (figure 5*a*–*e*). However, in such cases, as one species evolves towards maximization or minimization, the location of the CSS of the other species can change. On the other hand, if both trade-offs decelerate, we get either coevolutionary cycles or both the host and parasite evolve away from the cosingularity to reach the bounds of evolution (figure 5*a*–*e*).

We delve deeper into the impact of varying levels of virulence and crowding on the emergence of branching and cycles (figure 5*b*–*e*). Comparing the patterns with our default parameter values (*α* = 2, *q* = 0.5, figure 5*a*), we note that an increase in virulence from 1.5 to 3 results in a broader range where branching or cycles can occur (figure 5*b*,*c*). A similar trend is identified when examining the influence of the natural death rate (*b*) variation (see electronic supplementary material, appendix, figure 4*a*). On the other hand, as crowding factor *q* increases from 0.2 to 1.8, we observe a smaller branching region at *q* = 0.2, followed by an expansion at *q* = 0.5, and then a subsequent contraction with further increments in crowding (figure 5*a*,*d*,*e*). This suggests that a wider range of trade-off shapes allows branching or cycles at high virulence and intermediate crowding levels. In conclusion, our findings suggest that diversification is more likely at low or intermediate infected density, attributed to greater intrinsic or disease-induced deaths (high *b*/*α*) or intermediate crowding (*q*) in this coevolutionary model.

## 4. Discussion

Our modelling study has examined the coevolution of sterility tolerance in the host and transmission in the parasite, when traded-off with mortality tolerance and recovery rate, respectively. Our focus was on understanding how various factors such as ecological dynamics and trade-off shapes influence the outcomes. Our analysis yielded several important results, including: (i) co-CSS patterns corresponding to increasing virulence and natural death rate closely match the purely evolutionary CSS trends, but coevolution drives the parasite to lower transmission as crowding increases in contrast to when the parasite evolves alone, (ii) branching in host tolerance can occur, and can drive the parasite to also branch, (iii) cycles in the population dynamics can prevent coexisting dimorphic host and parasite strains from reaching their extremes, (iv) coevolutionary cycles can occur across a range of trade-off shapes and parameters, (v) branching is more likely for parameters corresponding to low/intermediate infected population densities.

Host–parasite coevolutionary models have demonstrated a variety of qualitative outcomes, such as stable investment in both host and parasite strategies (co-CSS), evolutionary branching in either or both species, coevolutionary cycles or maximization/minimization of evolving traits to their physiological bounds [3–6,8–10,14,16]. It is commonly believed that tolerance mechanisms do not result in evolutionary branching due to non-negative frequency dependence [28,29,55], although some exceptions exist [55,63]. However, Singh & Best [32] recently found that branching can occur in host sterility and mortality tolerance if both are directly correlated by a trade-off. To our knowledge, branching in host tolerance is not yet documented in any of the coevolutionary theoretical studies. Out of the few coevolutionary models looking at host tolerance, Best *et al.* [55] and Carval & Ferriere [64] demonstrated that coevolution alone cannot lead to genetic variation in host tolerance. Later, Best *et al.* [22] investigated the potential for diversity in mortality tolerance using coevolutionary frameworks and concluded that neither branching nor cycles could occur in either species. Our study reveals that when the parasite coevolves, the host can still branch with a sterility-mortality tolerance trade-off. In fact, it can cause the parasite to also branch, which does not occur when the parasite evolves in isolation. This aligns with the findings of a recent theoretical study exploring the coevolution of parasite virulence and host mortality tolerance through a defensive symbiont [65]. They discovered that the defensive symbiont can cause the parasite to split into two subpopulations, resulting in the coexistence of strains with low and high virulence. An experiment on *A. thaliana* subjected to *Turnip mosaic virus* (TuMV) and *Cucumber mosaic virus* (CMV) found that tolerance to TuMV was traded-off against tolerance to CMV in a virulence-dependent manner [35]. However, the authors found no evidence of a fecundity-mortality tolerance trade-off against a single virus and concluded that the host and virus genotypes play a significant role in such trade-offs. This calls for a theoretical and empirical investigation of our model in a multi-pathogen context. Furthermore, we found coevolutionary cycles (‘temporal’ rather than ‘static’ diversity), a key result which is also not yet observed in coevolutionary models of tolerance.

Interestingly, we found that the coexisting dimorphic strains that arise after branching do not evolve to their extremes, a departure from most of the existing theoretical studies [6,22], which appears to be driven by cycles in our population dynamics model. It is well established that population dynamics can create qualitative and quantitative effects on coevolutionary dynamics due to eco-evolutionary feedbacks [66], with precise effects depending on the model [1]. In particular, fluctuating population dynamics can impact the selection of evolving traits by affecting contact rates between hosts and parasites [56]. Theoretical models have examined the impact of fluctuating ecological dynamics on parasite and host evolution [63,67–70], but only one study investigated this for a host–parasite coevolutionary model [31]. A recent study serves relevant insights in this context, showing how the presence of population cycles can alter selection and how evolution can shift the population dynamics between cycles and ecological equilibria [57]. In our two host–two parasite population dynamics system, increasing differences between trait values in each species resulted in limit cycles, causing quantitative changes in coevolutionary outcomes. Interestingly, the system appeared to settle close to the boundary of cycles and equilibria. Testing how ecology and coevolution interact to drive host–parasite interactions remains a key area of theoretical research.

In theory, parasite evolution is primarily investigated through trade-offs between parasite-induced mortality (virulence) and transmission. This transmission-virulence trade-off hypothesis postulates that a more aggressive host exploitation strategy increases parasite transmission, but it also leads to higher virulence [71]. Studying the evolution of parasite sublethal effects, however, becomes difficult using this framework where negative effects of the parasite are only characterized as host mortality [38], yet many parasites are nonlethal or cause only moderately virulent infections [72]. With this in mind, Alizon [38] and Greischar *et al.* [40] have emphasized the significance of investigating links between transmission and recovery rate. Greischar *et al.* [40] found that higher transmission can lead to faster recovery from infection. This positive correlation between transmission and recovery (as immune clearance) has also been observed in data-based studies on *Plasmodium chabaudi* in laboratory mice [73] as well as in both rodent malaria and human malaria parasite, *P. falciparum* [74]. Given that this motivation for a transmission-recovery trade-off is partly due to the effects of the host’s immunological response, there may also be direct connections to host tolerance or defence mechanisms in general. Insights from experimental research could inform the development of future models incorporating these connections.

Our work highlights the significance of trade-off shapes in coevolution. Small changes in trade-off choice can drastically alter coevolutionary outcomes, as previously noted [50]. For example, changing fitness costs can result in the dynamics shifting between stable investment and fluctuating selection, as shown by previous studies [7,16,75]. The inherent complexity of coevolutionary models can make it challenging to make firm predictions on outcomes without precise knowledge of trade-offs, relative mutation speeds and trait values. Further, we found that results from stochastic simulations could vary slightly to the deterministic predictions, for example finding branching occurring in simulations when cycles were expected. Generally, while coevolutionary cycles can arise for various trade-off shapes, our model shows that stable branching in both species only occurs for a few trade-off curvatures.

One key aim of this study is to understand the distinctions in coevolutionary dynamics compared with single-species evolutionary models. Our coevolution model demonstrates branching in both host and parasite populations, whereas in isolation, it occurs only in the host [32]. We additionally discovered that a broader range of trade-off shapes facilitates diversification through branching or cycles for parameters associated with low or intermediate infected population densities (high intrinsic/disease-induced death rates and intermediate crowding). This stands in contrast to the outcomes of the host evolution models [32,63], where such diversification occurs at intermediate to high infected densities. Thus, our findings suggest that parasite coevolving can shift the coevolutionary dynamics and facilitate the possibilities of genetic variation. Furthermore, while results on the stable investments from the host-only evolution model matched our coevolutionary trends, there were differences in the pattern for varying crowding factor when only the parasite evolves. Specifically, varying crowding creates no impact on transmission when the parasite evolves in isolation but coevolution drives transmission to decrease as crowding increases. On the other hand, while prevalence exhibited a decreasing trend in response to both virulence and the crowding factor in the host evolution model [32], our model showed an initial rise, then decline, then another rise in prevalence with increasing virulence, and an overall increase with crowding. This highlights how coevolution can generate varied feedbacks on parasite prevalence within the host population.

This study examined the coevolution of a host with a trade-off between two tolerance mechanisms and a parasite with a transmission-recovery trade-off without assuming shared control over any particular epidemiological trait. Our model provides initial predictions on coevolutionary patterns, especially regarding coevolutionary branching and cycles in host tolerance. Our findings on genetic variation in tolerance traits leading to divergent parasite populations imply the need for experiments to confirm the existence of tolerance trade-offs in systems where such genetic diversity is apparent. Moreover, our result related to high prevalence in conditions of high crowding and heightened virulence underscores the necessity for experiments in controlled environments to predict the conditions leading to high disease prevalence. Studying allocation to tolerance mechanisms within natural systems, considering costs and parasite spread, can offer valuable insights for developing disease control strategies based on tolerance. While tolerance–tolerance trade-offs are important for understanding host defence evolution, our parasite trade-off hypothesis may have implications for anti-parasite treatments, as it allows the host to recover more quickly from infections. Implementing these epidemiological findings can improve predictions for how parasites evolve in response to treatments.

## Data Availability

Matlab codes for the simulations are available on Figshare [76].
